# Reliability and validity study of measurements on digital photography to evaluate shoulder balance in idiopathic scoliosis

**DOI:** 10.1186/s13013-014-0023-6

**Published:** 2014-12-14

**Authors:** Antonia Matamalas, Juan Bagó, Elisabetta D’Agata, Ferran Pellisé

**Affiliations:** 1 Department of Orthopaedic Surgery, Hospital Vall d’Hebron, P Vall d’Hebron, 119, Barcelona, 08035 Spain; 2 Research Institute, Hospital Vall d’Hebrón, P Vall d’Hebrón, 119, Barcelona, 08035 Spain

**Keywords:** Cosmetic, Idiopathic scoliosis, Photography, Shoulders balance

## Abstract

**Objective:**

To determine the validity of digital photography as an evaluation method for shoulder balance (ShB) in patients with idiopathic scoliosis.

**Material and methods:**

A total of 80 patients were included (mean age 20.3 years; 85% women). We obtained a full x-ray of the vertebral column and front and back clinical photography for all patients. For antero-posterior x-rays we measured the proximal thoracic curve angles (CPT). To evaluate radiological shoulder balance we calculated the clavicle-rib intersection angle (CRIA) and T1-tilt. For clinical photography we measured shoulder height angle (SHA), axilla height angle (AHA) and the left right trapezium angle (LRTA). We analyzed the reliability of the different photographic measurements and the correlation between these and the radiological parameters.

**Results:**

The mean magnitude of PTC, CRIA and T1-tilt were 19°, −0.6° and 1.4° respectively. Mean SHA from the front was −1.7°. All photographic measurements revealed an excellent-near perfect intra and inter-observer reliability in both photographic projections. No correlation was found between the ShB and the magnitude of the PTC. A statistically significant correlation was found between clinical balance of the shoulders and radiological balance (r between 0.37 and 0.51).

**Conclusions:**

Digital clinical photography appears to be a reliable method for objective clinical measurement of ShB. The correlation between clinical and radiological balance is statistically significant although moderate/weak.

**Electronic supplementary material:**

The online version of this article (doi:10.1186/s13013-014-0023-6) contains supplementary material, which is available to authorized users.

## Introduction

Cosmetic disorder is one of the main reasons to treat Idiopathic Scoliosis patients (SOSORT Consensus) [1]. Shoulder balance (ShB) has been considered a characteristic of the deformity in idiopathic scoliosis [2]-[4]. According to Raso, this represents 75% of the perceived deformity of the trunk, together with asymmetry of the scapulae and shoulder girdle [2].

To be able to evaluate this balance correctly, reliable tools are necessary. Different evaluation methods ranging from radiological, clinical and topographical have been proposed over the years. Hong et al. [5] recently evaluated the reliability and validity of the different radiological methods to evaluate ShB and concluded that, in general, all outcomes have reliable intra and inter-observer reliability. Nonetheless, radiological balance does not appear to optimally correspond with clinical balance, which suggests that clinical parameters should be a complement to radiological outcomes [6].

Different methods have been proposed to assess shoulder imbalance [2]-[4]. Zaina et al., have developed a tool for routine clinical use (TRACE), consisting of photographs depicting different severities of four aspects of trunk deformity; shoulder, scapulae, hemithorax and waist. This instrument relies on subjective impression of the observer [7]. Surface topometry such as Moiré-Fringe or 3D scan (Vitrus) [8] methods have been used. Nonetheless, these systems require expensive equipment and a trained operator, which means that their usefulness in clinical practice remains a moot point. Conversely, clinical photography corrects some of these defects: the equipment is cheap, simple to handle and quick to obtain. These reasons lead to supposing that it may be of considerable interest in daily practice.

Two indices have traditionally been used to study the shoulders in clinical photography; the shoulder height difference (in cm or by means of the angular measurement) and the axillary fold height difference [9]-[11]. Other indices to evaluate ShB have recently been proposed shoulder height difference at the level of the upper border of the trapezium muscle [6],[9],[11],[12] and the area of the trapezium muscles [12]. The problem with these latter indices lies in the fact that each author defines them differently. Furthermore, data on the reliability of these outcomes are incomplete because no inter-observer reliability data for any of them are available, especially for front-view photography. We think it is necessary to ascertain the validity of the measurements taken from the front because this view corresponds to the patient’s view when they look in a mirror.

The aims of our research are twofold: a. to determine the test-retest reliability of various clinical measurements taken with digital photography and to compare the data between front and rear shots; b. to determine the validity of these photographic measurements by analyzing their relationship with the radiological measurements of ShB.

## Materials and methods

This is a transversal study approved by the clinical research ethics committee of Hospital Vall d’Hebrón. The inclusion criteria for this study were patients with idiopathic scoliosis with a largest Cobb angle (MLC) greater than 25° Cobb in the coronal plane, aged between 12 and 40 who agree to take part in the study. Patients were recruited consecutively; only patients who had not received surgery were included. At the time the picture was taken, no patient was on an active treatment with brace. The sample was stratified according to MLC into two groups: Group <45° and Group ≥45°. This cut-off value of 45° was chosen because at this magnitude, surgical treatment can be recommended. For each patient there was a postero-anterior x-ray of the full trunk in standing position performed the week before taking part in the study.

### Radiological measurements

**Figure 1:**
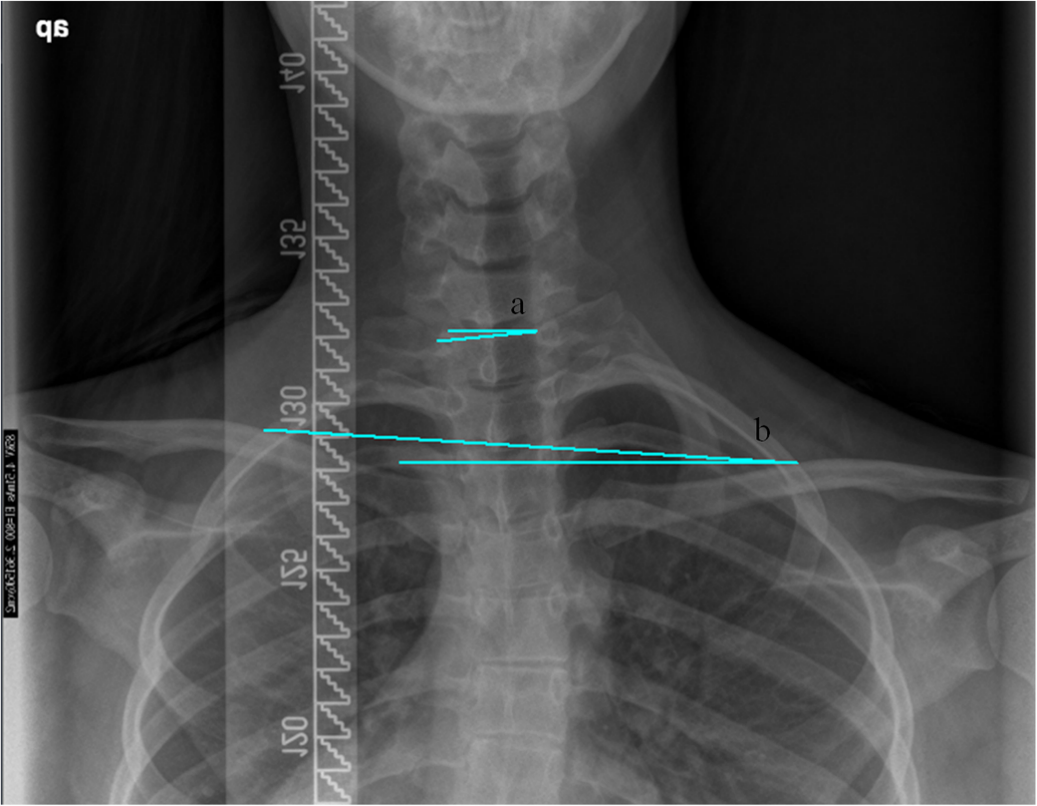
**Radiological measurements of shoulder balance.** **(a)** T1-tilt **(b)** Clavicle-rib intersection angle (CRIA).

**Figure 2:**
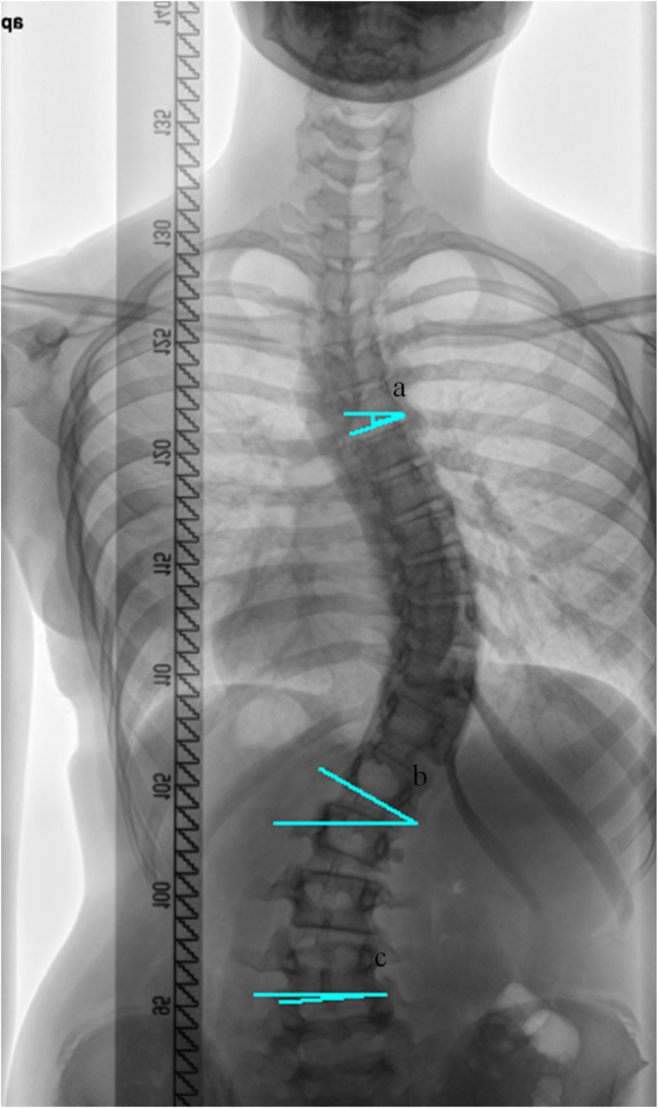
**Radiological measurements of end vertebra.** **(a)** Lower end vertebra of the proximal thoracic curve (PTC_LEV). **(b)** Lower end vertebra of the main thoracic curve (MTC_LEV). **(c)** Lower end vertebra of the thoraco-lumbar/lumbar curve (TLLC_LEV).

The following was recorded on postero-anterior x-ray: the magnitude of the proximal thoracic (PTC), main thoracic (MTC) and thoraco-lumbar/lumbar (TLLC) curves. Furthermore, the tilt with respect to the horizontal of T1 (T1-tilt) (Figure 1), the lower end vertebra of the PTC (PTC_LEV), the lower end vertebra of the MTC (MTC_LLV) and lower end vertebra of the TLLC (TLLC_LLV) (Figure 2), was measured. Radiological ShB was calculated by means of the Clavicle-Rib Intersection Angle (CRIA). CRIA is defined as the angle formed by the horizontal and a line that joins the intersection points of the clavicles with the rib-cage (Figure 1).

### Photographic measurements

Each patient underwent clinical photographs on the same day of the visit by just one trained examiner (EA) who undertook the entire process.

To acquire the photographs a digital Nikon D5100 (Nikon Corporation, Tokyo, Japan) camera, mounted on a tripod at 110 centimeters in height and with a distance of 130 centimeters, was used for both photographs. The patients’ position was standardized on a previously marked cross on the floor. Patients were told to adopt a relaxed standing position when the photographs were taken. All of them were photographed with an anterior (front) and posterior (back) view.

**Figure 3:**
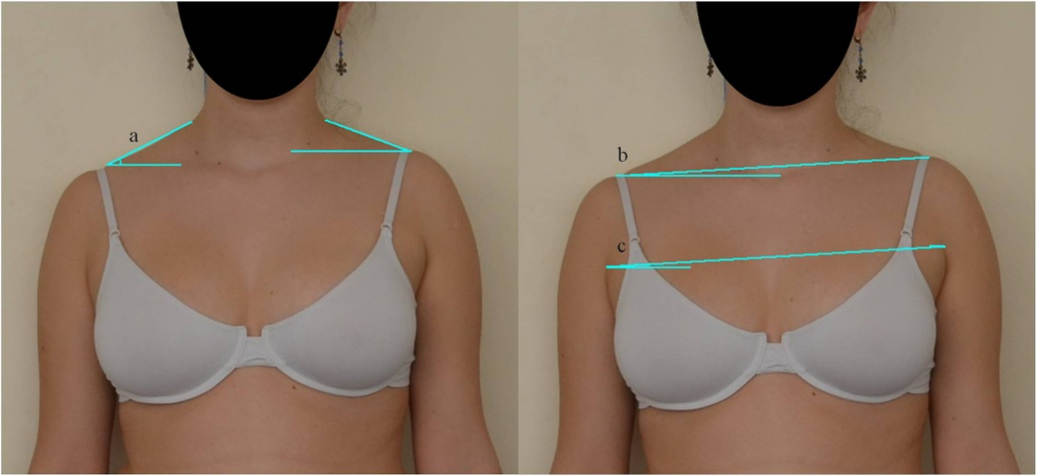
**Photographic measures in front view.** **(a)** Right and left trapezium angle. **(b)** Shoulder height angle. **(c)** Axilla height angle.

**Figure 4:**
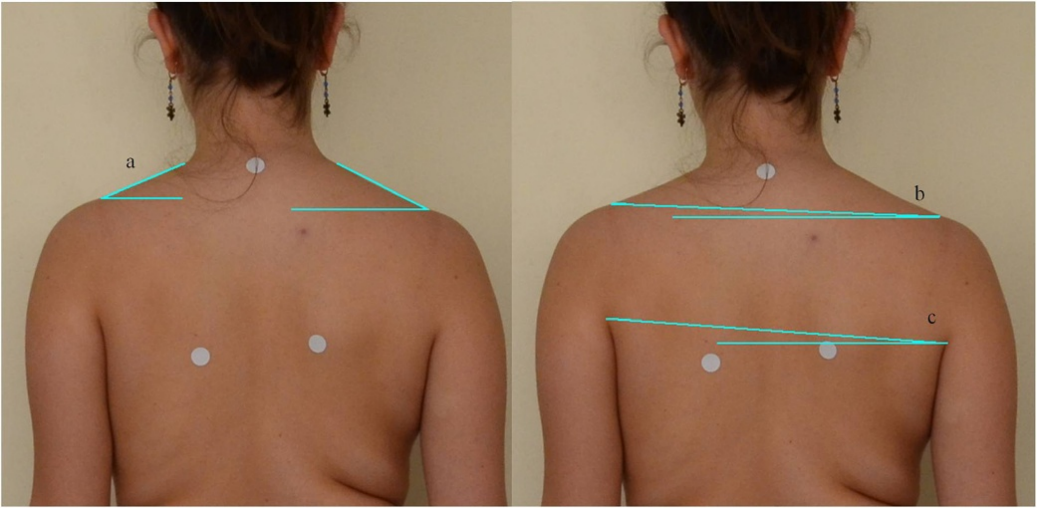
**Photographic measures in back view.** **(a)** Right and left trapezium angle. **(b)** Shoulder height angle. **(c)** Axilla height angle.

For each photograph three photographic indices were calculated:

*Left/right trapezium angle (LRTA)*: The trapezium angle is defined as the angle between the line following the external border of the trapezium muscle and the horizontal. The left/right ratio of this angle was used for statistical analysis (Figures 3 and 4).

*Shoulder height angle (SHA)*: Angle formed between the line that joins the upper border of both acromion processes and the horizontal (Figures 3 and 4).

*Axilla height angle (AHA)*: Angle formed between the line that joins the upper border of both external axillary folds and the horizontal (Figures 3 and 4).

The SurgimapSpine® (Nemaris Inc, New York, United States) software was used for both the x-ray and clinical photography measurements. Both the radiological and photographic measurements were assigned a positive or negative value according to the tilt direction. The right-hand thumb rule was used for this: looking at the individual (or x-rays) from the back, a clockwise and anti-clockwise tilt was considered positive and negative, respectively. For example, the tilt of the end vertebrae towards the right was assigned a positive value; elevation of the left shoulder was assigned a positive value; curves with convexity to the left and right were assigned a positive and negative value, respectively.

### Statistical analysis

Descriptive statistics (mean and range) were used to report patients and radiological and photographic measurements.

To determine the reliability of the photographic indices an intra and inter-observer reliability analysis using the intraclass correlation coefficient (ICC) with absolute agreement and a 95% confidence interval, was performed. To analyze reliability photographs of the first 60 patients (30 cases with MLC < 45° and 30 cases with MLC > 45°) were used. Each photograph was measured by three evaluators (AM, JB, EA) on two separate occasions, one week apart. To calculate intra-observer reliability all three observers’ measurements were analyzed (in total 180 measurements). The first and second measurements of all observers were jointly compared. To calculate inter-observer reliability the first measurement made by each observer was used. The intra and inter-observer intra-class correlation coefficient was obtained for each measurement. According to Landis & Koch [13] the following scale was used to interpret the ICC: <0.20 minimal or inexistent relationship; 0.21 - 0.40 poor; 0.41- 0.60 moderate; 0.61 - 0.80 excellent and >0.81 near perfect.

To study convergent validity, Pearson correlation coefficients between the photographic and radiological measurements were calculated.

To test the degree of equivalence of the back and front SHA and the degree of equivalence between the front SHA and AHA, the concordance correlation coefficient was determined [14],[15]. Similar to the Pearson coefficient, the CCC varies between −1 and +1. The criteria recommended by McBride were used to determine the degree of concordance: >0.99 Near perfect; 0.95-0.99 Substantial; 0.90-0.95 Moderate and < 0.90 Poor [16].

Data were processed using a SPSS 17.0 program. *P* < 0.05 was considered statistically significant.

## Results

### Sample descriptions

A total of 80 consecutive patients were included. Mean patient age (typical deviation) was 20.3 years (± 8.6) and 85% of patients were women. A total of 68.8% of patients had attained skeletal maturity (Risser 4 and 5) at the time of inclusion into the study and 16.3% were immature patients (Risser 1 and 2). The distribution of frequencies of the different kinds of curve according to the Lenke classification [17] was: type 1 (27.5%), type 2 (5%), type 3 (26.3%), type 4 (2.5%), type 5 (32.5%) and type 6 (6.3%). Table 1 shows the means and range of different radiological and photographic measurements.

**Table 1:** **Descriptions of the radiological and photographic outcomes**

Variables	Mean (°)	Range	
		Minimum	Maximum
**Radiological**			
PTC	18.9	−15.9	46.3
PTC_LEV	−17.4	−42	19.2
MTC	−33.5	−78.0	53.2
MTC_LEV	16.5	−35	42.1
TLLC	24.1	−55.6	60.9
TLLC_LEV	−7.7	−33.7	36.9
T1 tilt	1.4	−16.9	29.2
CRIA	−0.6	−12.1	7.3
**Photographic**			
LRTA back	1.1	0.7	2.0
SHA back	−0.7	−8.4	6.2
AHA back	−1.8	−8.2	7.9
LRTA front	1.2	0.6	3.4
SHA front	−1.7	−11.1	5.5
AHA front	−2.3	−11.9	6.4

PTC (Cobb proximal thoracic curve); PTC_LEV (Lower end vertebra of the proximal thoracic curve); MTC (Cobb main thoracic curve); MTC_LEV (Lower end vertebra of the major thoracic curve); TLLC (Cobb thoraco-lumbar/lumbar curve); TLLC_LEV (Lower end vertebra of the thoraco-lumbar/lumbar curve); T1 tilt (T1 inclination angle); CRIA (clavicle-rib intersection angle); LRTA (Left/right trapezium angle ratio); SHA (Shoulder height angle); AHA (Axilla height angle).

### Reliability and standard error of measurement

Table 2 represents intra and inter-observer reliability values and the standard error of measurement for the different photographic measurements. As can be seen in the table, the intra-observer ICC values for SHA and AHA both front and back were >0.80 indicating a near perfect correlation; for LRTA the intra-observer ICC were excellent (0.79 and 0.78 respectively). The inter-observer ICC were slightly less although within the near perfect correlation range (> 0.80) except for the front LRTA which was excellent (0.65).

**Table 2:** **Intra and inter-observer reliability and standard error of measurement (SEM) of the photographic measurements**

Variable	Intra-observer reliability (ICC)	Inter-observer reliability (ICC)	Standard error of measurement (SEM)
**Back**			
LRTA	0.79	0.80	0.14
SHA	0.88	0.80	0.99
AHA	0.93	0.88	0.83
**Front**			
LRTA	0.78	0.65	0.24
SHA	0.91	0.89	1.06
AHA	0.91	0.85	1.18

LRTA (Left/right trapezium angle ratio); SHA (Shoulder height angle); AHA (Axilla height angle).

### Concordance

The SHA and AHA angles in the photograph taken frontally presented poor concordance (CCC 0.66; 95% CI = 0.53-0.76). The same occurs with the SHA angles for front and back whose concordance was also poor (CCC 0.49; 95% CI = 0.32-0.64).

### Correlations between photographic and radiological measurements

Table 3 lists the correlation coefficients between the photographic and radiological measurements. The correlation of photographic measurements with radiological parameters was poor to moderate. Only PTC correlated poorly (r = −0.26) with AHA for the back shot; there was no correlation with any other photographic parameter. For MTC we observed a moderate correlation (r = 0.46) with AHA posterior view; there were poor correlations with the frontal view clinical parameters (r ranging from −0.27 to 0.27. TLLC was moderately to poorly correlated with all photographic parameters both for front and back view (r ranging from −0.45 to 0.35). The end vertebrae of the different curves revealed similar correlations to those of the overall magnitude of the curve.

**Table 3:** **Correlation between the clinical outcomes of imbalance of the shoulders and the radiology**

Correlations												
	Back						Front					
	LRTA		SHA		AHA		LRTA		SHA		AHA	
	***r***	***p***	***r***	***p***	***r***	***p***	***r***	***p***	***r***	***p***	***r***	***p***
CRIA	−0.35	0.00	0.39	0.00	0.35	0.00	−0.45	0.00	0.48	0.00	0.51	0.00
T1	−0.35	0.00	0.37	0.00	0.31	0.005	−0.35	0.00	0.51	0.00	0.44	0.00
PTC	−0.07	n.s.	0.03	n.s.	−0.26	0.02	−0.07	n.s.	0.04	n.s.	−0.03	0.02
MTC	−0.16	n.s.	0.19	n.s.	0.46	0.00	−0.27	0.02	0.24	0.03	0.27	0.01
TLLC	0.35	0.002	−0.33	0.003	−0.45	0.00	0.23	0.04	−0.26	0.02	−0.30	0.007
PTC_LEV	−0.11	n.s.	0.17	n.s.	0.45	0.00	−0.27	0.15	0.25	0.02	0.29	0.008
MTC_LEV	0.19	n.s.	−0.19	n.s.	−0.42	0.00	0.24	0.29	−0.21	n.s.	−0.24	0.03
TLLC_LEV	−0.44	0.00	0.39	0.00	0.36	0.001	−0.15	n.s.	0.25	0.02	0.28	0.01

*LRTA (Left/right trapezium angle); SHA (Shoulder height angle); AHA (Axilla height angle);* CRIA (Clavicle-rib intersection angle); T1 tilt (T1 inclination angle); *PTC (Proximal thoracic curve Cobb angle); MTC (Main thoracic curve Cobb angle); TLLC (Thoraco-lumbar/lumbar curve Cobb angle);* PTC_LEV (*Lower end vertebra of the proximal thoracic curve*); MTC_LEV (*Lower end vertebra of the major thoracic curve*); TLLC_LEV (*Lower end vertebra of the thoraco-lumbar/lumbar curve*).

The radiological measurement of ShB (CRIA) showed moderate correlation with the photographic measurements, especially the frontal photograph (LRTA r = −0.45; SHA r = 0.48 y AHA r = 0.51). A statistically significant correlation was found between the photographic measurements and T1-Tilt, especially for the SHA in the frontal view (r = 0.51).

## Discussion

Shoulder balance is considered characteristic of idiopathic scoliosis. Use of clinical photography to measure ShB has not been fully analyzed. Clinical photography offers a series of practical advantages: it is cheap, simple to handle and images are almost immediately available. The aims of our research were to determine the reliability of various measurements taken using digital photography and to evaluate their relationship with radiological parameters in a non-selected population of patients with idiopathic scoliosis.

### Selection of measurements

An initial step to design the research was to decide which measurement to include in the study. It was decided to select angular measurements to avoid problems from the calibration necessary when using linear measurements. In the case of asymmetry of the trapezium muscles, it was preferred to use angular measurements instead of surface areas as we believe that the latter is more complex and not very useful in daily clinical practice. We also ruled out using skin markers as other authors had done previously. We believe that this methodology lengthens examination time and introduces a new source of bias. We therefore preferred to define, a priori, the anatomic points to be used as a reference for the measurements.

After these prior considerations, it was decided to record three parameters:

1. Shoulder height angle (SHA): formed between the line that joins the upper border of both acromion processes and the horizontal. This is the parameter which should, a priori, collate shoulder imbalance better. This parameter has been used previously by other researchers both for front [9],[12] and back [6],[9],[12] photography. Some authors [18] have used the linear measurement by calculating the difference in cm from the upper border of each acromion process to a horizontal line perpendicular to the axillary fold. This methodology requires calibration, whereby it was rejected. Furthermore, for SHA we only have reliable data from back photography [11].
2. The left/right ratio trapezium angle (LRTA) reported as the angle formed by the external border of the trapezium muscle with the horizontal. We think this could be equivalent to the Ln [L/R Trapezium Area] reported by Ono [12]. These authors found a statistically significant correlation between this parameter and the radiological variables. Nonetheless, there are no reliable data for this measurement and, in our opinion; its calculation is excessively complex for routine use. The possibility of recording an evaluation parameter for the trapezium area was put forth by prior publications which indicate its relationship with the proximal thoracic curve.
3. Axilla height angle (AHA): formed between the line that joins the upper border of both acromion processes and the horizontal. This parameter has also been used previously [6],[11]. It was decided to include it to analyze its possible relationship with radiological shoulder imbalance with the intention of having a second parameter to estimate shoulder imbalance for those cases when SHA is not reliable. For AHA we only have reliable data from the measurement during back photography [11].

### Reliability and concordance

Most of the measurements selected revealed excellent-near perfect intra and inter observer reliability (ICC > 0.70); the inter-observer ICC were slightly less, a data already reported by other authors [19]. The reliability data are very similar for frontal and back views. Yang et al. reported somewhat more reliability (intra-observer reliability 0.97 for both measurements and inter-observer reliability 0.99 and 0.97 respectively) for the back photography [11]. The intra and inter-observer reliability values for LRTA, SHA and AHA from the front, used in our work, have not been previously published.

We found poor concordance between SHA and AHA, which suggests that one measurement cannot estimate another when analyzing the clinical balance of the shoulders. Similarly, when evaluating the concordance between front and back SHA we found poor concordance between both measurements (CCC 0.49, 95% CI 0.32-0.64); this indicates that both measurements are not interchangeable between themselves.

### Relationship between the photographic and radiological measurements

Overall, the correlations found between clinical and radiological parameters may be considered moderate to poor and in no case greater than 0.6. Behavior was similar for the three parameters evaluated (SHA, AHA and LRTA) for the two photographic views (front and back). This low correlation is similar to that reported when analyzing correlations between the radiological parameters and those obtained with the topographic analysis technique [20].

From our results, the lack of correlation between clinical ShB and magnitude of the PTC curve is notable; because it is usually accepted, that one of the factors that has an impact on shoulder imbalance, is the structural nature of this curve. Other previous publications had found poor or inexistent correlations between radiographic and photographic measurements in type 1 and 2 Lenke curve series [6],[9],[12],[21]. These data suggest that the PTC does not have a significant impact on clinical shoulder balance. Conversely, we have found a moderate correlation between MTC and TLLC and the photographic measurements of the ShB, especially with the back AHA (r = −0.44). Yang and Qiu found a similar correlation [6],[9] and Hong et al. reported that post-operative ShB in a series of patients who had received surgery, was related to the correction of the MTC and TLLC [22]. These findings would indicate that clinical ShB would in part be influenced by the magnitude of the main thoracic curve and the lumbar curve. The tilt of the end vertebrae for the different curves correlated in a similar way to those overall values of the curves with the photographic measurements. No especially interesting correlation was found; therefore, the vertebra to vertebra analysis does not appear to be useful. Overall, the parameters measured in the frontal view reveal correlations with the radiographic measurements somewhat higher than those found for the rear view. Specifically, we have to point out the correlation between SHA and CRIA (r = 0.48) and SHA and T1-tilt (r = 0.51). Therefore, we would venture to recommend that the study of ShB be performed on photography taken from a frontal view, although we are aware that this shot may be a reason for conflict or rejection, especially in the case of women.

The photographic parameters (SHA, AHA, LRTA) were moderately correlated with CRIA and T1 tilt. We hypothesize that CRIA would be the radiological equivalent of SHA. Different parameters were used for the radiological measurement of ShB: Coracoids’ height difference (CHD) [5],[21],[23], clavicular angle (CA) [5],[23], clavicle-rib intersection difference (CRID) [23], radiological shoulder height (RSH) [5],[23] or first rib angle (FRA) [9],[12] among others. Our initial intention was to use clavicular angle (CA) as a radiological measure of ShB considering the high level of reliability of the measurement reported by Hong et al. [5]. Nonetheless, we find that for a high percentage of patients both shoulders on the x-rays could not be observed. For this reason, we decided to use the point where the clavicle crosses the ribcage as a reference point. Bagó et al. [23] found an excellent correlation between the difference in real shoulder height and that measurement at this reference point.

The correlation between SHA and CRIA was less than expected taking into account the fact that, theoretically, both measurements evaluate the same feature. In our study, no correlations between the two parameters greater than 0.54 were found. Other authors have found similar correlations between these measures when evaluating Lenke 1 and 2 curves [9]. This low correlation cannot be attributed to the reliability of the parameters evaluated if we consider that in all works published the reliability of the photographic measurements is excellent [6],[11] and the same occurs with radiological measurements [5]. It is possible that the photographic measurements differ from radiological measurements because of the effect of the soft tissues in the shoulder area. It is obvious that the radiological and clinical balance of the shoulders are not an exact reflection of each other as suggested by Qiu et al. [6]; we need to evaluate both factors when analyzing shoulder balance in patients with scoliosis, not just on the Lenke 2 curves but also for all kinds of curves.

T1-tilt moderately correlates with the photographic parameters (SHA, AHA, LRTA). Therefore, shoulder position cannot be inferred from a T1 value. In fact, there is a percentage of patients in whom shoulder and T1 tilt are in opposite directions [24]. Other authors have found that the correlation of this measure with shoulder balance, both radiological [23] and clinical [18],[21] is lower than for other measures such as CA or CRID. Bearing in mind that T1 is often the upper end vertebrae of the PTC and that the magnitude of the PTC is unrelated to ShB, our data indicate that T1 tilt should be the criterion to determine the structural nature of the PTC and its impact on ShB.

SHA can be considered the standard parameter to evaluate ShB in clinical photography. There is suitable intra and inter-observer reliability although the correlation with its radiographic equivalent is less than desirable. AHA is also a reliable measure but has a low correlation with radiological ShB. It is interesting to note the moderate correlation with the magnitude and tilt of the end vertebrae of the MTC which would suggest that this would be a parameter more related to deformity of the trunk than ShB. As we have pointed out above, this parameter was introduced to explore the possibility of having an alternative measure to SHA. The lack of concordance between both measures has led us to rule out this possibility. The possibility that LRTA would enable evaluating PTC led us to introduce this parameter into the analysis. In spite of correct reliability, this only shows a poor correlation with CRIA and T1 and no correlation with PTC. Although other authors have suggested that asymmetry in the trapezium area is a parameter to consider when clinically evaluating the shoulder area [12], according to our results, this is a parameter that does not provide information for SHA and AHA. Consequently, we do not believe that it makes sense to recommend use of this parameter in clinical practice.

### Shortcomings

In our opinion this study presents several significant limitations. First, our study did not include analysis of the photographic parameters in relation to the scoliosis pattern. Some authors [11] have suggested that the photographic parameters could be different according to the type of curve. This possibility should be analyzed in further detail in future investigations. Second, we have not correlated ShB and axial plane deformity (angle of trunk inclination or apical vertebrae rotation); we take this decision due to the low reliability of radiographic measures used for this purpose [25]. Third, a single photograph evaluated by different observers on two occasions was used for the reliability analysis. However, the reliability of this shot was not determined. Patients were placed on floor marks and they were asked to stay in a comfortable position. We think that this methodology was sufficient to guarantee repeating the photograph. However, we cannot determine the error of measurement related to the patient’s position. Fortin et al. found significant reliability of a photography technique similar to that used in our investigation [10],[19].

## Conclusions

Clinical photography is a reliable method to evaluate clinical shoulder balance in patients with idiopathic scoliosis. Intra and inter-observer reliability is excellent; ICC greater than 0.8 were found. The reliability of the front and back views is similar although concordance analysis reveals that the measurements are not equivalent. These data confirm that ShB is not a pathognomonic sign of structured scoliosis. Based on the present results, the measurement of SHA does not seem an appropriate method to evaluate the effect of treatment on spinal deformity. Consequently, both examinations should be used for shoulder balance evaluation. In the future, it should be analyzed whether shoulder imbalance pattern varies according to curve pattern.

Written informed consent was obtained from the patient for the publication of this report and any accompanying images.

## Authors’ original submitted files for images

Below are the links to the authors’ original submitted files for images. Authors’ original file for figure 1
Authors’ original file for figure 2
Authors’ original file for figure 3
Authors’ original file for figure 4

## Abbreviations

ShB: Shoulder balance
PTC: Cobb proximal thoracic curve
MTC: Cobb main thoracic curve
TLLC: Cobb thoracic-lumbar/lumbar curve
MLC: Major large Cobb
T1-Tilt: Inclination of T1
CRIA: Clavicle-rib intersection angle
LRTA: Left-right trapezium angle
SHA: Shoulder height angle
AHA: Armpit height angle
ICC: Intra-class correlation coefficient
SEM: Standard error of measurement
MDC: Minimal detectable change
CCC: Concordance correlation coefficient
CHD: Coracoids’ height difference
CA: Clavicle angle
RSH: Radiological shoulder height
FRA: First rib angle
PTC_LEV: Proximal thoracic curve lower end vertebra
MTC_LEV: Main thoracic curve lower end vertebra
TLLC_LEV: Thoraco-lumbar/lumbar lower end vertebra
