# Supplementary figures and images for: Reliability and validity study of measurements on digital photography to evaluate shoulder balance in idiopathic scoliosis
Source: Scoliosis. 2014 Dec 14;9:23. doi: 10.1186/s13013-014-0023-6 (PMC4269069; doi:10.1186/s13013-014-0023-6)

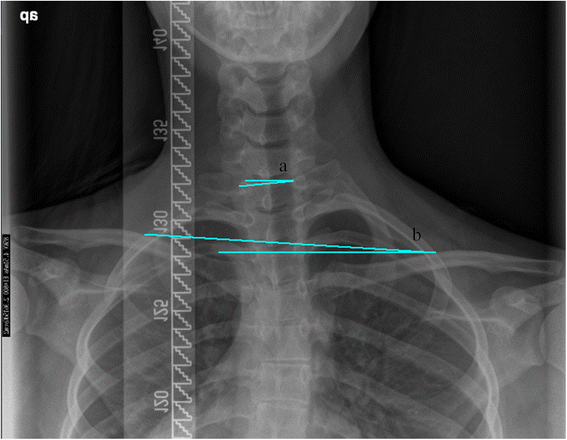

Supplement: Supplementary file 1 — Authors’ original file for figure 1 [file 13013_2014_23_MOESM1_ESM.gif]

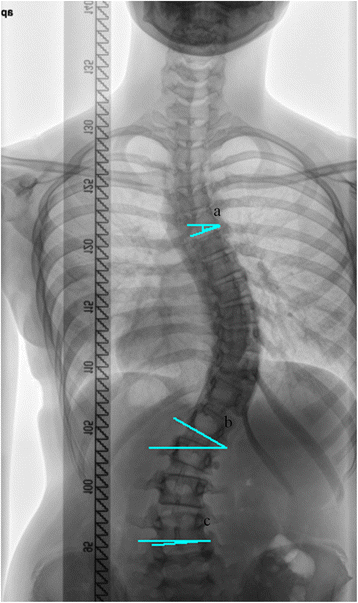

Supplement: Supplementary file 2 — Authors’ original file for figure 2 [file 13013_2014_23_MOESM2_ESM.gif]

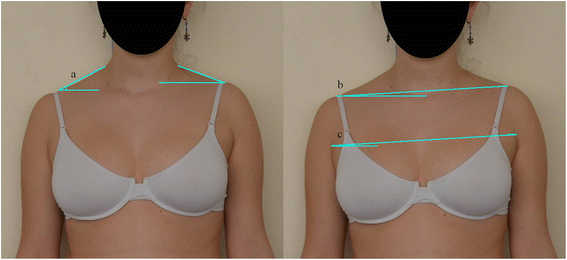

Supplement: Supplementary file 3 — Authors’ original file for figure 3 [file 13013_2014_23_MOESM3_ESM.gif]

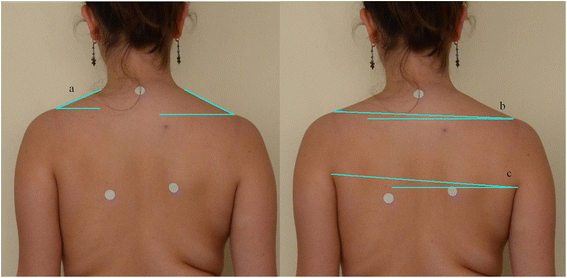

Supplement: Supplementary file 4 — Authors’ original file for figure 4 [file 13013_2014_23_MOESM4_ESM.gif]
